# Effect of Hyperoxia on Retinoid Metabolism and Retinoid Receptor Expression in the Lungs of Newborn Mice

**DOI:** 10.1371/journal.pone.0140343

**Published:** 2015-10-28

**Authors:** Hsing-Jin Chen, Bor-Luen Chiang

**Affiliations:** 1 Graduate Institute of Clinical Medicine College of Medicine of National Taiwan University, Taipei, Taiwan; 2 Graduate Institute of Immunology, National Taiwan University, Taipei, Taiwan; Medical University of South Carolina, UNITED STATES

## Abstract

**Background:**

Preterm newborns that receive oxygen therapy often develop bronchopulmonary dysplasia (BPD), which is abnormal lung development characterized by impaired alveologenesis. Oxygen-mediated injury is thought to disrupt normal lung growth and development. However, the mechanism of hyperoxia-induced BPD has not been extensively investigated. We established a neonatal mouse model to investigate the effects of normobaric hyperoxia on retinoid metabolism and retinoid receptor expression.

**Methods:**

Newborn mice were exposed to hyperoxic or normoxic conditions for 15 days. The concentration of retinol and retinyl palmitate in the lung was measured by HPLC to gauge retinoid metabolism. Retinoid receptor mRNA levels were assessed by real-time PCR. Proliferation and retinoid receptor expression in A549 cells were assessed in the presence and absence of exogenous vitamin A.

**Results:**

Hyperoxia significantly reduced the body and lung weight of neonatal mice. Hyperoxia also downregulated expression of RARα, RARγ, and RXRγ in the lungs of neonatal mice. *In vitro*, hyperoxia inhibited proliferation and expression of retinoid receptors in A549 cells.

**Conclusion:**

Hyperoxia disrupted retinoid receptor expression in neonatal mice.

## Introduction

Bronchopulmonary dysplasia (BPD) is a chronic lung disease that often develops in premature infants who require mechanical ventilation with oxygen [1]. The pathophysiological changes of BPD include inflammation, airway fibrosis, and smooth muscle hypertrophy, which can result in poor alveolarization and disrupted microvascular development [2]. Almost one-quarter of extremely low birth weight infants (<1500 g) develop BDP [3]. BPD remains a serious challenge in caring for premature infants. Hyperoxia-induced BPD is characterized by the accumulation of inflammatory cells, and production of inflammatory cytokines and chemokines in the lung [4]. The underlying mechanism of hyperoxia-induced BPD is an active area of research, but remains incompletely understood [5]. A better understanding of the molecular mechanisms that lead to hyperoxia-induced BPD could provide insights for developing novel therapeutic and preventive strategies that can be used during oxygen therapy.

An active area of research is the role of inadequate nutrition in the pathogenesis of BPD. Notably, vitamin A deficiency plays a key role in BDP development [6]. Vitamin A (retinol) is a member of the retinoid family, a group of molecules including retinol, retinoic acid (an active metabolite of retinol), and retinaldehyde [7]. Vitamin A has important functions in epithelial cells and contributes to their differentiation and helps maintain the integrity of the epithelial barrier, especially the epithelial cells lining the respiratory tract [8]. BPD also shares several characteristics with vitamin A- deficient patients such as atelectasis, loss of ciliated cells, keratinizing metaplasia, and necrotizing bronchiolitis [9–11]. Consistent with this observation, preterm infants with BPD often have lower plasma and liver retinol levels than preterm infants without BPD [12–13]. In addition, previous studies have suggested that vitamin A supplements can reduce the risk of BPD, chronic lung disease of prematurity [14], and the morbidity associated with BPD [15]. Retinoic acid has been shown to have numerous benefits in the lung in rodent models including aiding alveologenesis in neonatal rat models [16], relieving symptoms of pulmonary emphysema in rats [17–19], attenuating hyperoxia-induced impaired alveolar development in mice [20], and attenuating the inhibitory effects of glucocortisteroids and hyperoxia on septation in newborn rats [21]. Taken together, these observations suggest that retinoic acid plays a crucial role in normal lung development and that retinoic acid deficiency could contribute to the pathogenesis of BPD.

As key components of the retinoid-signaling pathway, retinoic acid receptors are also implicated in neonatal lung development [22,23]. There are two types of retinoid receptors, retinoic acid receptors (RARs) and retinoid X receptors (RXRs). RAR and RXR each have three known isoforms designated α, β, and γ [24,25]. Upon binding to their retinoid ligands, RARs form heterodimers with RXRs [26] and act as ligand-activated transcription factors to activate target gene expression [27]. During embryonic development, retinoid receptors play critical roles in organogenesis [28]. For example, several isoforms of RAR and RXR are upregulated during the pseudoglandular phase in the fetal mouse lung [29,30]. Several genetic studies have described the functions of the RARs and RXRs in the fetal mouse lung. Lung hypoplasia was observed in RXRα/RARα and RARα/β double knockout mice [31,32], and RARγ knockout mice have tracheal cartilage malformations [33]. These genetic studies clearly demonstrate that retinoid receptors play a role in early lung development and alveolarization. However, the effects of hyperoxia on the expression of genes for RARs and RXRs are still largely unknown.

To further investigate the mechanism of hyperoxia-induced BPD, we examined the effects of hyperoxia on cell proliferation, retinoid metabolism, and expression of retinoic acid receptors in mouse and cell models. We hypothesized that hyperoxia would alter the metabolism of retinoids and the expression of retinoid receptors.

## Materials and Methods

### Animals and care

Timed-pregnant Imprinting Control Region (ICR) mice were used since female ICR mice give birth to approximately 16–20 pups in a single gestation, which were sufficient for our experiments. ICR mice were obtained from the Laboratory Animal Center, College of Medicine, National Taiwan University (NTU). The mice were housed under standard laboratory conditions with food and water ad libitum and kept in a 12 h light-dark cycle in the accredited Laboratory Animal Center, College of Medicine, NTU. The experimental protocol was approved by the NTU Hospital Medical Center Animal Care and Use Committee.

### Induction of hyperoxia

Within 12 h of birth, pups from three to four litters were pooled and randomly redistributed between the newly delivered mothers. Half of the newborn mice were housed under hyperoxic conditions with 90% oxygen, while the other half were housed in 21% oxygen and served as the normoxic control group. The litters were reduced to 10–12 per nursing mother to ensure the same nutrients receiving in all groups. Nursing mothers were rotated between hyperoxic and control litters every 24 h to avoid oxygen toxicity in the mothers and to eliminate maternal effects between groups. To establish hyperoxic conditions oxygen was continuously delivered at 2.5–3.0 L/min into chambers housing the mice. The continuous flow achieved a constant level of 90% oxygen and prevented CO_2_ accumulation. Oxygen levels were monitored with a Miniox II monitor (Catalyst Research, Owings Mills, MD). Survival was recorded daily, and mice were kept in hyperoxic conditions for up to 15 d. The body weight was recorded at the time of death.

### Tissue preparation

Mice were sacrificed at P1, P4, P9, and P15 by intraperitoneal injection of pentobarbtol sodium (200 mg/kg). The lungs and livers were removed during dissection. Lung and liver tissues for real-time PCR analysis were removed, washed with phosphate-buffered saline (PBS), and frozen in liquid nitrogen, then immediately stored at -80°C until the RNA was isolated.

### Real-time PCR analysis

Real-time PCR was used to describe the temporal expression of retinoic acid receptor isoforms during hyperoxia. RNA was extracted using a Qiagen RNAeasy kit, and cDNA was prepared using an Amersham first-strand cDNA synthesis kit, as described in the manufacturer's instructions. RNA extractions were performed using lungs from at least four mice in each group and each lung was analyzed separately. The following specific primers and probes were purchased from ABI (USA) and used to amplify each gene of interest: murine RARα (Mm00436264_ml), RARβ (Mm01319676_ml), RARγ (Mm01296255_ml), RXRα (Mm00441182_ml), RXRβ (Mm00441193_ml), RXRγ (Mm00436411_ml), and human RARα (Hs00940453), RARγ (Hs01559230_m1), and RXRγ (Hs009760000). The amplification conditions were: denaturation for 30 s at 95°C, annealing for 30 s at 55°C, and extension for 30 s at 72°C. Quantitative real-time PCR analysis was carried out using the ABI 7900 system. Each experiment was repeated at least three times with similar results.

### Determination of retinyl palmitate and retinol concentrations in the lung and liver

Liver and lung samples from neonatal mice were weighed and then homogenized and the organic compounds were extracted from the tissue using standard chloroform/methanol procedures as previously described [23,28]. The tissue extracts were dehydrated under nitrogen at 30°C and then solubilized in MeOH:CHCl_3_ (4:1). Aliquots of the extracts were assayed by reverse phase HPLC, using a pecosil C18, 5 μm, 15 cm column at a wavelength of 325 nm. All samples were protected from light at all times. The concentrations of retinol and retinyl palmitate were determined in duplicate by HPLC using a programmable liquid chromatographic system (Waters Associates, Milford, MA). The mobile phase for retinyl palmitate and retinol was 100% methanol. Standard curves were generated based on peak height using the same instrument settings as the assay conditions against known amounts of retinol and retinyl palmitate. The tissue concentration of each compound was expressed per unit wet weight.

### Immunohistochemistry

For immunocytochemistry, the lungs were exposed by thoracotomy, the trachea was cannulated, tied firmly in place with a scalp vein set (25- gauge blunt needle) connected to an infusion set and then vertically elevated to a distance of 20 cm, and infused with ice-cold 10% buffered formalin at pressure of 20 cmH_2_O. After 15 min, the trachea was ligated and the lungs and heart were removed en bloc and fixed with 10% buffered formalin overnight at 4°C. The lobes were then dehydrated in graded ethanol, cleared in xylene, and embedded in paraffin. Paraffin sections 4 μm thick were deparaffinized with xylene and rehydrated using graded ethanol and water. Cell proliferation was evaluated by immunostaining for the proliferation maker Ki67 (1:50; Dako Cytomation, Carpenteria, CA). Briefly, following rehydration, tissue sections were microwaved for 20 min in citrate buffer. Endogenous peroxidase was quenched using 3% hydrogen peroxide in methanol and then washed in TBS buffer. The slides were rinsed in TBST, blocked for 30 min using Rodent Block Mixture (Biocare Medical, USA), and then incubated over night at 4°C with 1:50 anti-Ki67 in 0.1% PBS-Tween 20 and 2% horse serum. Antigen was detected with a biotinylated secondary horse anti-mouse antibody followed by ABC Elite diaminobenzidine (DAB), according to the manufacturer’s directions. The slides were then counterstained with hematoxylin. The negative controls were tissue samples that were incubated with PBS/FCS rather than primary antibody.

### Quantitative Immunohistochemistry

Proliferation was quantified in lung tissue from five random noncontiguous fields of lung parenchymal/distal air space saccules from at least four separate animals in each group. Fields that contained a large airway or blood vessel were not used for the analysis. Each field was photographed at 40-fold magnification using a Zeiss Axiophot microscope (Zeiss, Oberkochen, Germany) and AGFA RSX II FILM (Agfa-Gevaert, Leverkusen, Germany; ASA 50). Quantification was performed using Metamorph software (Universal Imaging, Downingtown, PA). Metamorph was configured to measure the total number of nuclei based on the average area of a nucleus. The total number of cells that stained positive for Ki67 were counted manually using Metamorph to mark the counted cells. The total number of nuclei and the number of positively stained nuclei in each field were determined; 400–600 nuclei per field were counted for each tissue section. The frequency of Ki67+ cells was calculated as a percentage of the total cell nuclei in the alveolar (cells lining the distal air sacs and alveoli) and bronchiolar epithelial cells for each treatment group (n = 4–5 mice/group).

### Cell culture

A549 lung cancer cells were purchased from ATCC (USA). The cells were incubated in 5% CO2 at 37°C in RPMI supplemented with 5% fetal bovine serum (FBS), 50 U/mL of penicillin, and 50 μg/mL of streptomycin and L-glutamine. Cells were passaged twice before being used in experiments.

### Establishing hyperoxia in A549 cells

A549 cells were plated in 100-mm dishes at a density of 5x10^5^ cells/mL.The cultures were given 10 mL of fresh medium and placed in a sealed Plexiglas box (Belco Glass,Vineland, NJ). The cells were cultured in a humidified incubator chamber under normobaric hyperoxic conditions (95% oxygen- 5% CO_2_). The oxygen concentration was monitored with a MiniOxI system (Mine Safety Appliances Co, Pittsburgh, PA, USA).

### Vitamin A treatment in A549 cells

Under hyperoxic or normoxic conditions, A549 cells were cultured in RPMI media supplemented with all-*trans*-retinoic acid (Sigma, St. Louis, MO, USA). Retinoic acid was added to the culture at the same time cells were placed in hyperoxic conditions. The cells were exposed to normoxia or hyperoxia for 2 d and 3 d. After 3 d, the cells were harvested and gene expression and cell proliferation were assessed. In preliminary experiments, all-*trans*-retinoic acid was tested at concentrations of 10^−6^ to 10^−4^ mol/L. Subsequent experiments used retinoic acid at 10^−5^ mol/L because it resulted in the best cell viability during hyperoxic injury. We also tested the toxicity of the organic solvents used for preparing vitamin A, and did not observe any toxic effects.

### Thymidine[3H] incorporation in A549 cells

Proliferation of A549 cells was assayed using thymidine [^3^H] incorporation as an indicator of DNA synthesis. The cells were plated at a final concentration of 1x10^4^ cells per well in a 96-well plate in media containing 5% FBS with or without vitamin A. Each sample was tested in triplicate. Cells were then placed in a humidified chamber under normoxic (95% air-5% CO_2_) or hyperoxic (95% oxygen- 5% CO_2_) conditions for 2 d and 3 d. Thymidine [^3^H] was added for 18 h and then the cells were harvested. The amount of thymidine [^3^H] incorporated was measured using the Matrix 96 Direct beta Counter (Packard Instruments, Meriden, CT).

### Statistical analysis

Statistical analyses were done with IBM SPSS Version 20 (SPSS Statistics V20, IBM Corporation, Somers, New York). Continuous data were expressed as means ± standard deviation (SD). All group means within 2 groups were compared by Student’s t-test and over 2 groups by ANOVA. If overall ANOVA reached significance, Fisher's LSD as post-hoc procedure would be utilized to compared the difference between groups. Differences were considered significant at *p* < 0.05.

## Results

### Hyperoxia significantly inhibited the growth and survival of neonatal mice

**Fig 1:**
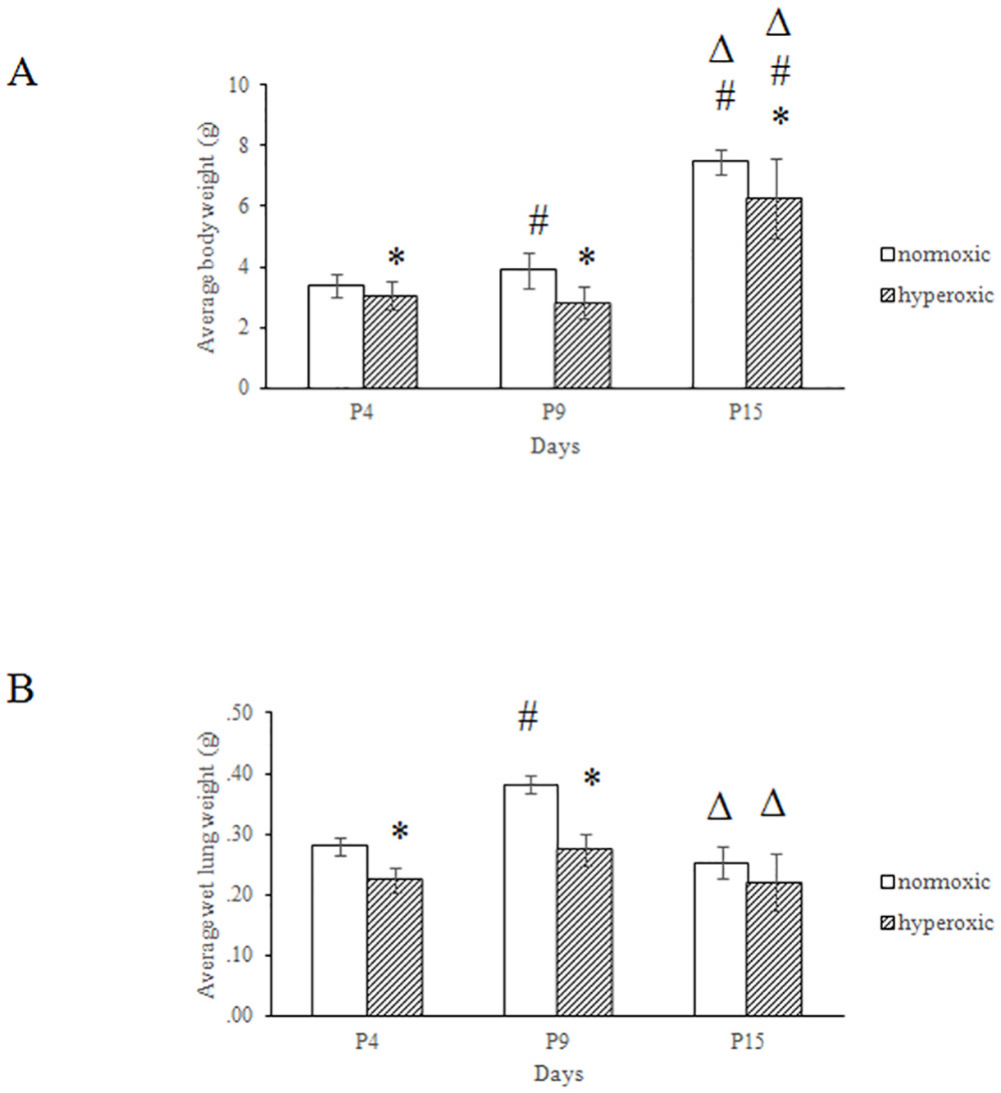
Hyperoxia significantly inhibited the growth of neonatal mice. Newborn mice were housed under either hyperoxic (90% oxygen) or normoxic (21% oxygen) conditions. Body weight (A, n = 12–20) and lung weight (B, n = 4) were recorded at the time of death. *P<0.05, vs. normoxic group at same time, ^#^P<0.05, vs. P4 group with the same treatment, ^Δ^P<0.05, vs. P9 group with the same treatment *<0*.*05*, all statistical comparisons by one-way ANOVA with Fisher's LSD as post-hoc procedure.

A neonatal mouse model was used to determine the effect of hyperoxia on lung development. The growth and survival of neonatal mice over 15 days was examined after exposure to hyperoxic conditions (92–95% O_2_) or normoxic (21% O_2_) conditions. Fifty-four percent of hyperoxia-treated mice survived, with the majority of deaths occurring between the 9^th^ (P9) and 15^th^ (P15) days of life (data not shown). As shown in Fig 1A, hyperoxia significantly inhibited the growth of neonatal mice. The hyperoxia-treated mice weighed an average of 9.9% less at P4, 27.5% less at P9, and 16.3% less at P15 than the control mice reared under normoxic conditions. Similar results were observed in terms of the net lung weight from mice in the two groups. The net lung weight of the hyperoxia-treated group was significantly lower than the normoxic controls by an average of 19.6%, 28.1%, and 12.9% at P4, P9 and P15, respectively (Fig 1B). These results demonstrated that hyperoxia significantly inhibited the survival rate, growth, and lung development of neonatal mice.

### Hyperoxia inhibited cell proliferation in the lungs of newborn mice

**Fig 2:**
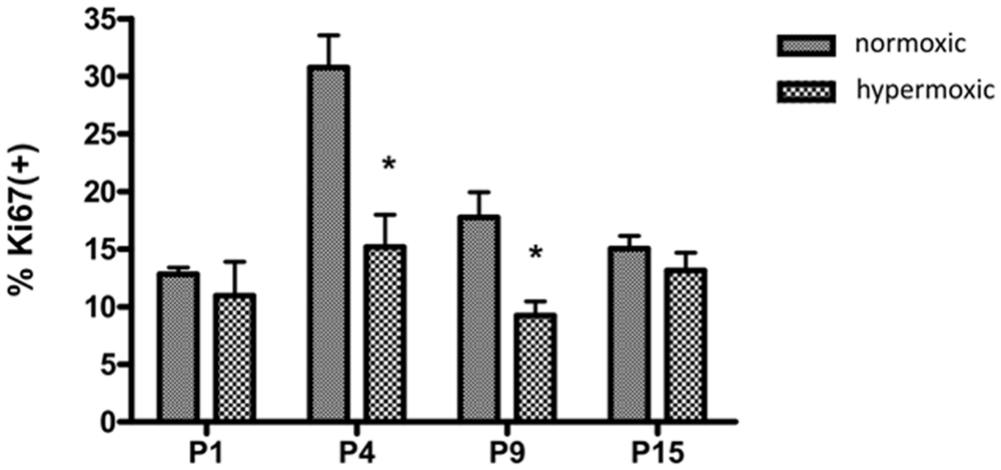
Hyperoxia inhibited cell proliferation in the lungs of newborn mice. Cell proliferation in the lung was evaluated by immunostaining for the marker Ki67. Five random noncontiguous fields of lung parenchymal tissue or distal air space saccules from at least 4 separate animals from each group were sampled. Each field was photographed at 40-fold magnification. Quantification was performed using Metamorph software. The frequency of Ki67+ cells was calculated as a percentage of the total alveolar (cells lining the distal air sacs and alveoli) and bronchiolar epithelial cells (n = 4–5mice/group). *p <0.05 vs. normoxic group, all statistical comparisons by one-way ANOVA with Fisher's LSD as post-hoc procedure.

One possible explanation for the reduced lung weight is decreased proliferation in lung epithelial or mesenchymal cells. To determine whether the lung weight loss was associated with decreased cell division, proliferation of lung cells was measured using Ki-67. Ki-67 was used to identify dividing cells and the frequency of Ki67+ cells was calculated in hyperoxia-treated and normoxic newborn mice. As shown in Fig 2, the frequency of Ki67+ cells was significantly decreased at P4 and P9 (*p* <0.05) in hyperoxic mice as compared with the normoxic controls, demonstrating that hyperoxia inhibited cell proliferation in the lungs of newborn mice.

### The effect of hyperoxia on the metabolism of retinyl palmitate in the lungs and livers of newborn mice

**Fig 3:**
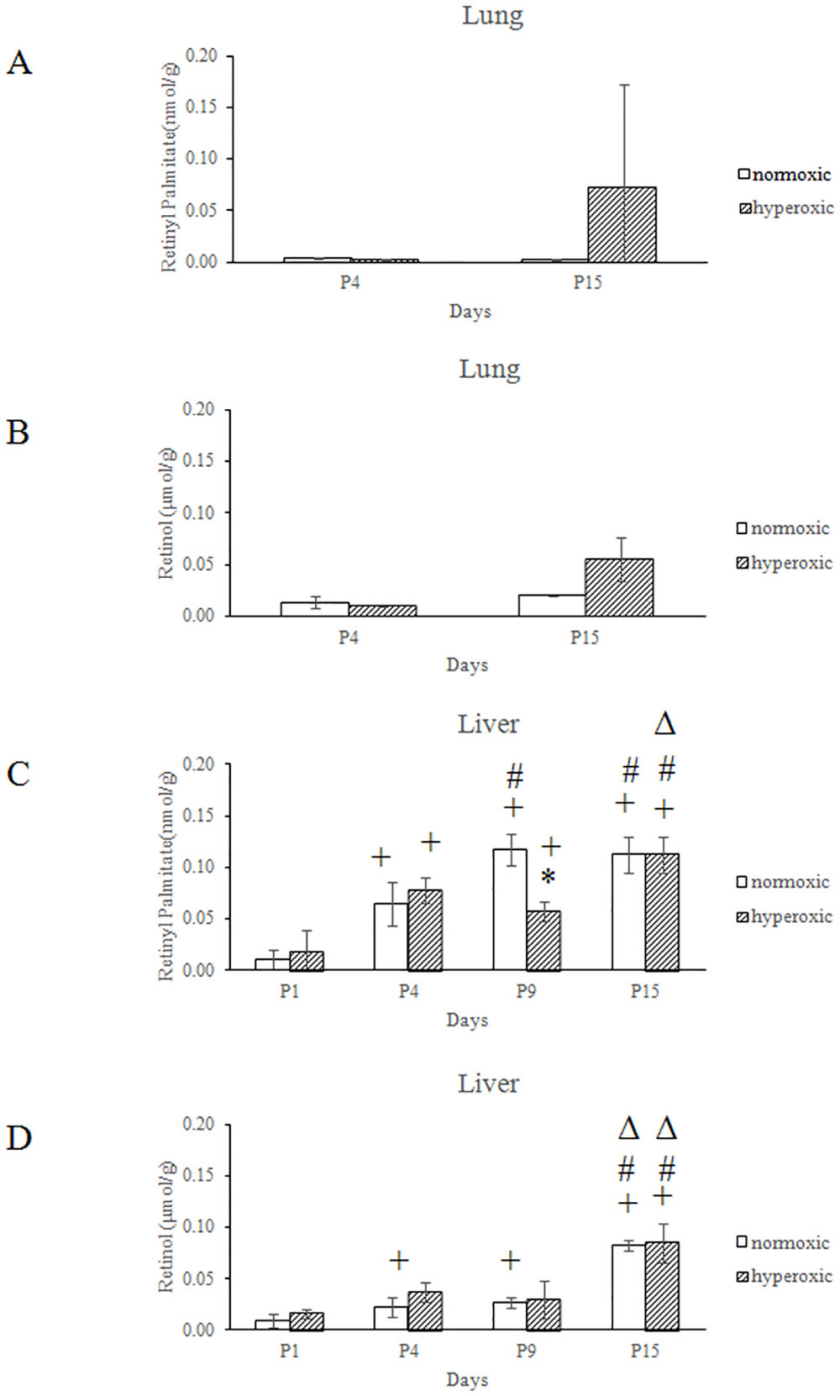
The effect of hyperoxia on the metabolism of retinyl palmitate. The concentration of retinol and retinyl palmitate was determined in the lungs (A, B) and livers (C, D) of newborn mice using HPLC. The HPLC assay used a pecosil C18, 5 μm, 15 cm column at a wavelength of 325 nm. The mobile phase for retinyl palmitate (RP) was (MeOH: CHCl_3_: H_2_O) (80:18: 2) at a flow rate of 1.5 mL/min, with a peak retention time of 5.5 min. *P<0.05, vs. normoxic group at same time, ^+^P<0.05, vs. P1 group with the same treatment, ^#^P<0.05, vs. P4 group with the same treatment, ^Δ^P<0.05, vs. P9 group with the same treatment, all statistical comparisons by one-way ANOVA with Fisher's LSD as post-hoc procedure.

We next addressed whether hyperoxia affected the metabolism of vitamin A. HPLC was used to measure the concentration of retinol and retinyl palmitate in the lungs and livers of neonatal mice. Retinoid extractions were performed on 4–6 replicate samples. All-*trans*-retinol and retinyl palmitate were detected in every sample, although the concentration varied according to the time after birth. As shown in Fig 3A and 3B, the concentration of retinyl palmitate and retinol in the lungs of newborn mice in the hyperoxia-treated group was higher, but not significant than the normoxic group at P15. Only a small amount of free retinol was detected in the lung over 15 days in both groups. In contrast, the mean concentration of retinyl palmitate in the livers of the hyperoxia-treated group was significantly decreased as compared with the control group at P9 (Fig 3C). There were no significant differences between the mean concentration of free liver retinol in the hyperoxia-treated group and control group (Fig 3D).

### The effect of exogenous vitamin A on hyperoxic injury in A549 cells

**Fig 4:**
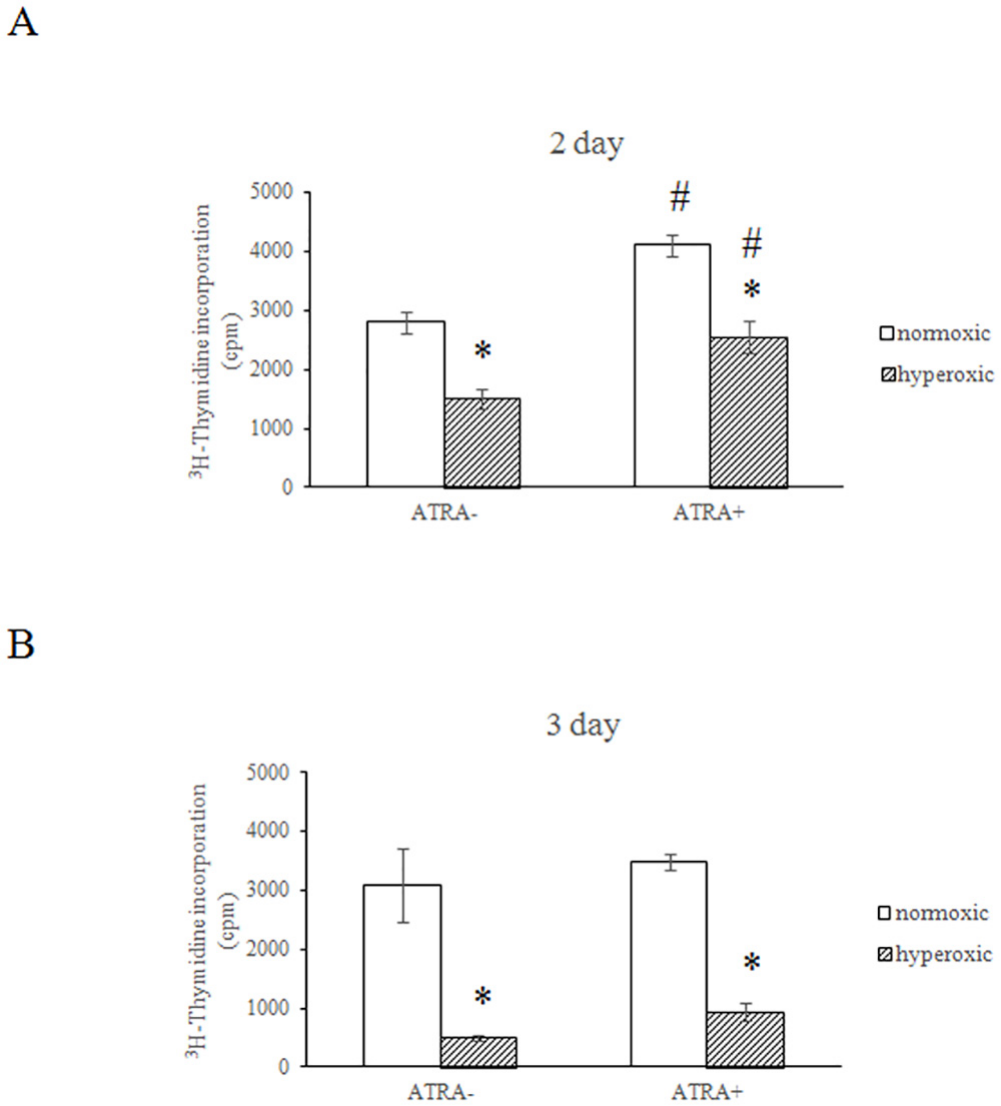
The effect of Vitamin A on the hyperoxia-induced decline of thymidine incorporation in A549 cells. Proliferation of A549 cells was assayed by thymidine [^3^H] incorporation, which measures DNA synthesis. The cells were plated at a final concentration of 1x10^4^ cells per well in a 96-well plate with or without vitamin A. Triplicates were tested for each sample. Cells were then placed in a humidified chamber with either 95% air-5% CO_2_ or 95% oxygen- 5% CO_2_ and cultured for 2 (A) or 3 d (B). Thymidine [^3^H] was added for another 18 h and then the cells were harvested and measured by Matrix 96 Direct beta Counter. *P<0.05, vs. normoxic group at same time, ^#^P<0.05, vs. ATRA- group with same treatment, all statistical comparisons by one-way ANOVA with Fisher's LSD as post-hoc procedure.

We next examined whether vitamin A could attenuate hyperoxic damage, using the A549 lung cancer cell line. As shown in Fig 4A and 4B, the thymidine incorporation in hyperoxia treated cells was significantly reduced as compared with their normoxic counterparts among all groups. Vitamin A was able to prevent the hyperoxia-induced decline of thymidine incorporation in A549 cells at day 2 (Fig 4A, *p* <0.05).

### RAR and RXR mRNA expression is elevated during lung development

**Fig 5:**
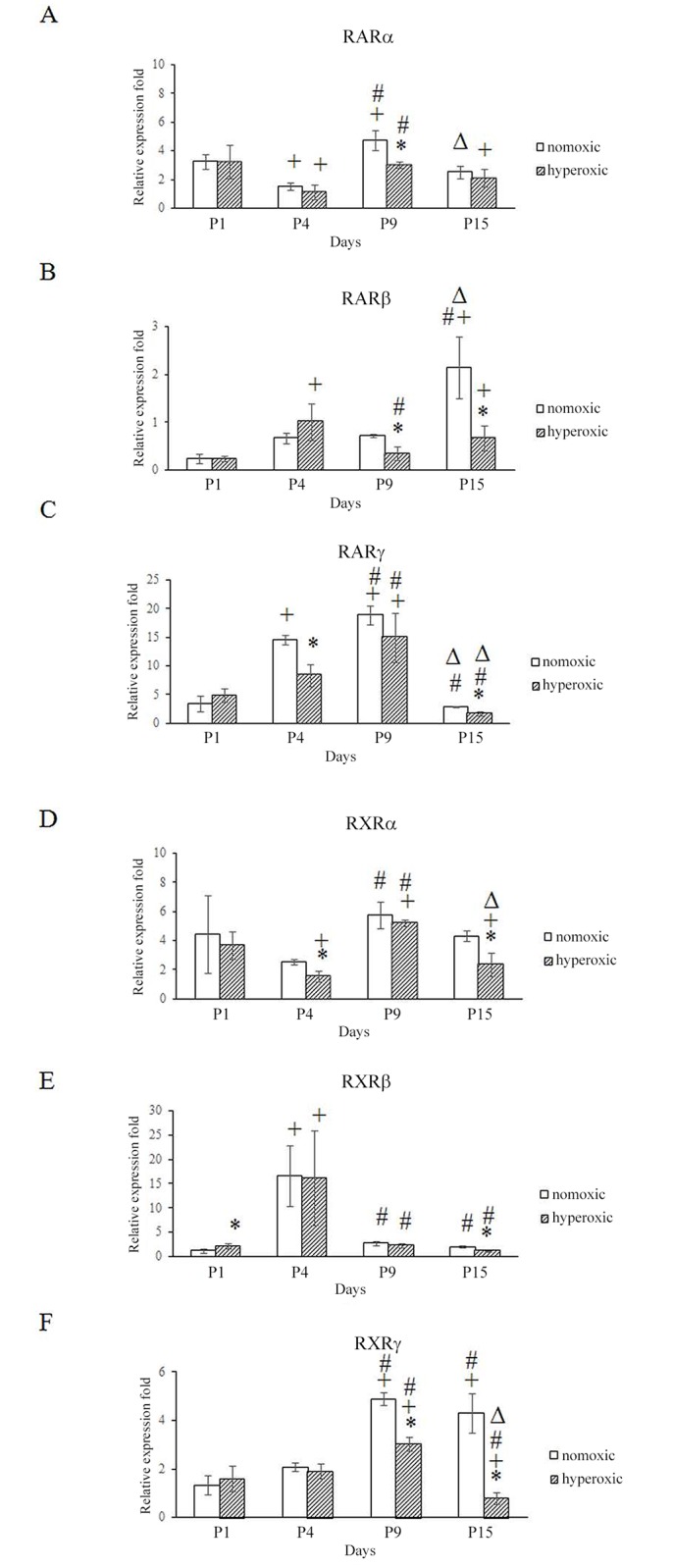
Hyperoxia altered retinoid receptor expression in the lungs of newborn mice. The mRNA expression of retinoic acid receptor isoforms in the lung was determined by real-time PCR, normalized to a *GAPDH* internal control, and presented as the fold difference in relative expression. Each experiment (n = 4) was repeated at least three times with similar results. <0.05<0.05 *P<0.05, vs. normoxic group at same time, ^+^P<0.05, vs. P1 group with the same treatment, ^#^P<0.05, vs. P4 group with the same treatment, ^Δ^P<0.05, vs. P9 group with the same treatment, all statistical comparisons by one-way ANOVA with Fisher's LSD as post-hoc procedure.

We hypothesized that hyperoxia might affect the expression of retinoid receptors. First, we examined the expression of RAR and RXR mRNA during normal postnatal lung development in neonatal mice. RAR (α, β, γ) and RXR (α, β, γ) mRNA expression was detectable and highly upregulated in the early postnatal mouse lung from P1 to P15 during alveolar septation (Fig 5). As compared with their expression on P1, RXRβ expression peaked at P4 (*p* <0.05), while RARβ expression peaked at P15 (*p* <0.05). The mRNA expression of three of the retinoid receptors, RARα (*p* <0.05), RARγ, and RXRγ (*p* <0.05) peaked at P9 as compared with their expression at P1. Based on the pattern of lung growth (Fig 1B), upregulation of retinoid receptor mRNA expression was consistent with alveolar septation in the postnatal mouse lung.

### Hyperoxia altered RAR and RXR expression in the lung

After confirming the expression profiles of retinoid receptors in normal postnatal mouse lung, we tested whether hyperoxia influenced the expression of retinoid receptors in the postnatal mouse lung. Mice reared under hyperoxic conditions had significantly reduced expression in five of the six RAR and RXR genes as compared with mice reared under normoxic conditions (Fig 5). However, the genes were not all suppressed at the same time. RARα was only significantly reduced at P9 (*p* <0.05); RXRα and RARγ were significantly reduced at P4 and P15 (*p* <0.05); and RARβ (*p* <0.05) and RXRγ (*p* <0.05) were significantly reduced on P9 and P15. Thus, hyperoxia altered the normal expression of RAR and RXR retinoid receptors.

### Hyperoxia-induced inhibition of RAR expression *in vitro*

**Fig 6:**
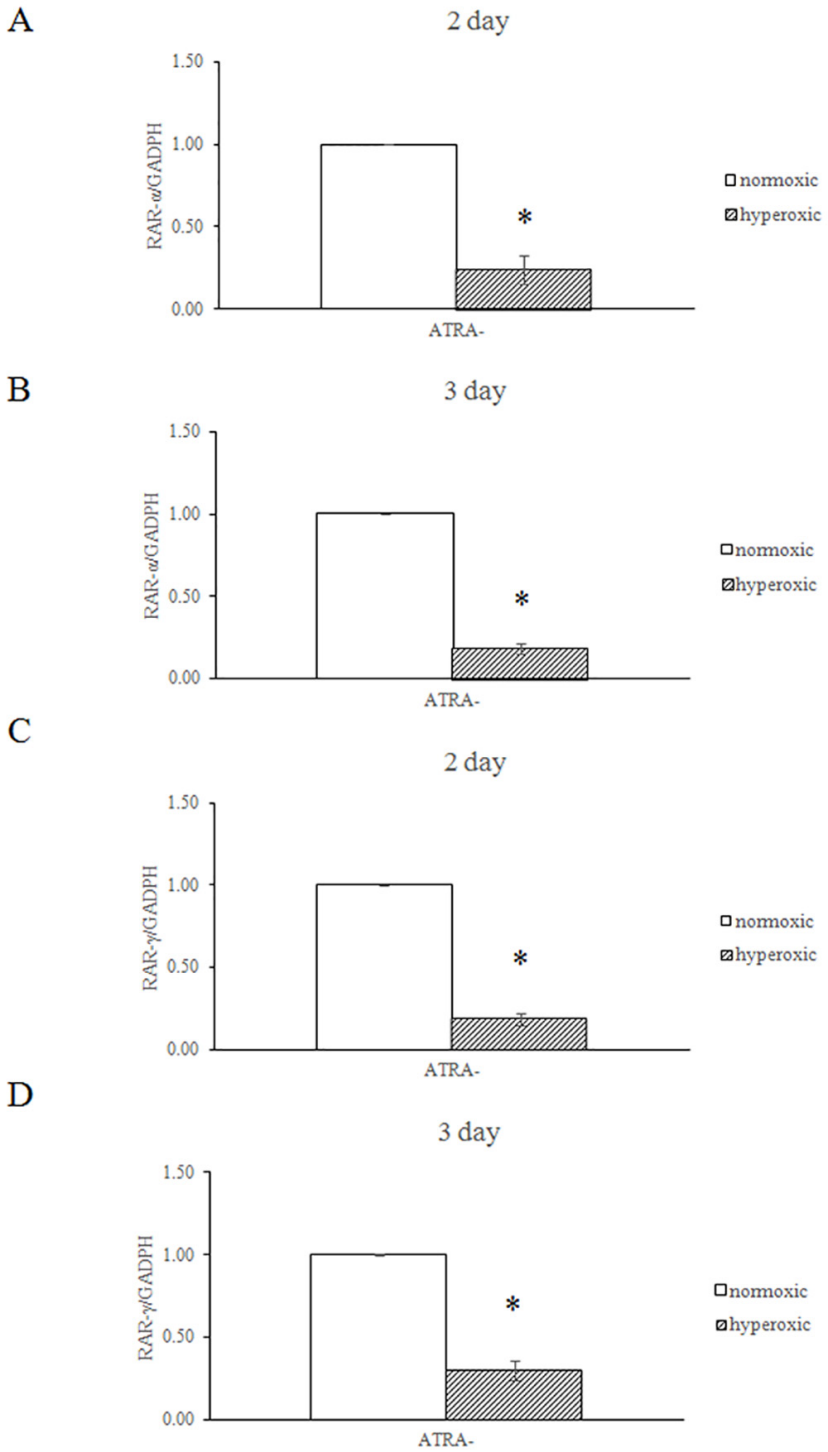
Hyperoxia-induced inhibition of RAR expression *in vitro*. A549 cells were plated in 100-mm dishes at a density of 5x10^5^cells/mL. The cells were then cultured in a humidified chamber under normoxic (95% air-5% CO_2_) or hyperoxic (95% oxygen- 5% CO_2_) conditions for 2 or 3 d. The cells were then harvested and assayed for RARα and RARγ expression. The expression levels were normalized to a *GAPDH* internal control. *P<0.05, vs. normoxic group, all statistical comparisons by one-way ANOVA with Fisher's LSD as post-hoc procedure.

The A549 cell culture model was used to test the effect of hyperoxia on RAR expression. As shown in the Fig 6, RARα and RARγ mRNA expression were significantly decreased in hyperoxia-treated cells as compared with normoxic controls.

## Discussion

Hyperoxia is a powerful pro-inflammatory stimulator; its toxic effects are mediated through reactive oxygen species (ROS). Hyperoxia activates a panel of pro-inflammatory cytokines, including IFNγ and macrophage inflammatory protein 2. Given their anti-inflammatory properties, antioxidants have been suggested as preventative and therapeutic options for BPD because they could protect the lung from damage by ROS and inflammatory cells [5]. Several reports have shown that retinol can attenuate hyperoxia-induced lung injury [34,35]. *In vitro*, we demonstrated that vitamin A treatment attenuated hyperoxia-induced growth inhibition in A549 cells at day 2, but it is still possible that hyperoxia reduced ATRA-induced A549 proliferation. The protective function of vitamin A has also been demonstrated in animal studies [21]. Hyperoxia is thought to inhibit lung cell proliferation by modulating p21-mediated cell cycle regulation, which can be prevented by pretreatment with retinoic acid [36, 37]. Our data showed that ATRA stimulated A549 proliferation at day 2 (but not day 3) under normoxic conditions, which contrasts to previous study [38]. The discrepancy might be attributed to different dose and time course of ATRA treatment. However, the exact molecular mechanism remains to be comprehensively investigated.

In this study, the mice maintained under hyperoxic conditions were significantly stunted with regard to body weight and lung development. Lung cell proliferation in newborn mice was significantly inhibited by hyperoxic conditions from P4 to P9, which is the stage when the most rapid proliferation occurs in the lung of newborn mice. This result demonstrated that the rapid growth phase of lung cells was most susceptible to hyperoxic exposure. These data were consistent with previous study [39]. In mice raised under normoxic conditions, the expression of RARα (P9), RARβ (P15), RARγ (P4 to P9), RXRβ (P4), and RXRγ (P9 to P15) increased significantly, which is consistent with the timeline of rapid lung cell proliferation in newborn mice. In contrast, under hyperoxic conditions, the expression of the 6 retinoid receptors were significantly reduced at P4 (RARγ, RXRα), P9 (RARα, RARβ, RXRγ), and P15 (RARβ, RARγ, RXRα, RXRβ, RXRγ), which could interfere with alveologenesis and contribute to BPD development. To our knowledge, this is the first study to report the expression patterns of retinoic acid receptors during hyperoxia. Our *in vitro* cell culture model showed that RARα and RARγ mRNA expression was inhibited by hyperoxia (Fig 6), which further supported that hyperoxia reduced the RARα and RARγ mRNA expression in neonatal mouse lungs. Additional experiments are required to clarify the molecular mechanisms of hyperoxia mediated inhibited the expression of retinoic acid receptor genes.

We also demonstrated that hyperoxia altered the metabolism of retinoids in the liver of newborn mice. After 15 days, the retinol concentration in the liver of hyperoxic mice was similar to controls reared under normoxic conditions. However, the concentration of retinyl palmitate, the stored form of retinol, in the liver was significantly decreased at P9 in hyperoxic mice. Our data was inconsistent with James ML *et al*.’s finding, which showed that a lower lung retinol concentration could be observed after mice exposed to hyperoxia14 days as compared with normoxic controls [20]. The discrepancy might be attributed to the differences in animal strain and the oxygen concentration of hyperoxia between two studies. Further study is required to clarify this discrepancy. In a hyperoxic environment, large quantities of ROS would be introduced into the microenvironment. As a result, antioxidants such as retinoic acid, retinol, and retinyl palmitate would be rapidly consumed to mitigate oxidative stress, especially in lung. The altered levels of retinyl palmitate and retinol in the liver of hyperoxia treated mice suggested that hyperoxia may have an effect on the metabolism of retinoid. It is worthy to conduct additional experiments to investigate the detailed mechanism.

This study provided a partial mechanism that could explain hyperoxia-induced BPD development. To our knowledge, we are the first to show that hyperoxia could interfere with the expression of retinoid receptors, which is critical for lung development. However, the molecular mechanism remains to be elucidated. There are some limitations of this study. The protective effects of retinol in terms of hyperoxia-induced growth inhibition and retinoid receptor expression remain to be confirmed in the animal model. The hyperoxia-mediated inhibition of retinoid receptors expression in lung also remains to be proved in the protein level. In addition, the intracellular signal transduction pathway underlying hyperoxia-induced RAR and RXR downregulation is still unclear. These limitations should be addressed in the following study.

In summary, we showed that hyperoxia could reduce retinoid receptor expression simultaneously, which might contribute to the pathogenesis of BPD.

## Supporting Information

S1 Table The statistical analysis methods and the detailed p values for all Figs. (XLSX)
